# Clinical practice guidelines standardisation of immunosuppressive and anti-infective drug regimens in UK paediatric renal transplantation: the harmonisation programme

**DOI:** 10.1186/s12882-021-02460-5

**Published:** 2021-09-16

**Authors:** Jan Dudley, Martin Christian, Alice Andrews, Nicola Andrews, Julie Baker, Sheila Boyle, Mairead Convery, Fiona Gamston, Martin Garcia, Shuman Haq, Shivaram Hegde, Richard Holt, Helen Jones, Shakeeb Khan, Jennifer McCaughan, David Milford, Charlie Pickles, Ben Reynolds, Vijaya Sathyanarayana, Jelena Stojanovic, Yincent Tse, Dean Wallace, Grainne Walsh, Nick Ware, Alun Williams, Pallavi Yadav, Stephen Marks

**Affiliations:** 1 Consultant Paediatric Nephrologist, Bristol, UK; 2 Consultant Paediatric Nephrologist, Nottingham, UK; 3 Lay Representative, Nottingham, UK; 4 Paediatric Renal Pharmacist, Birmingham, UK; 5 Transplant Nurse, Great Ormond St, London, UK; 6 Consultant Paediatric Nephrologist, Belfast, Northern Ireland; 7 Transplant Nurse, Birmingham, UK; 8 Specialist trainee in Paediatric Nephrology, Evelina, London, UK; 9 Consultant Paediatric Nephrologist, Southampton, UK; 10 Consultant Paediatric Nephrologist, Cardiff, UK; 11 Consultant Paediatric Nephrologist, Liverpool, UK; 12 Consultant Paediatric Nephrologist, Evelina, London, UK; 13 Consultant Transplant Surgeon, Bristol, UK; 14 Consultant Nephrologist, Belfast, Northern Ireland; 15 Consultant Paediatric Nephrologist, Birmingham, UK; 16 Specialist trainee in Paediatric Nephrology, Manchester, UK; 17 Consultant Paediatric Nephrologist, Glasgow, UK; 18 Consultant Paediatric Nephrologist, Newcastle, UK; 19 Consultant Paediatric Nephrologist, Great Ormond St, London, UK; 20 Consultant Paediatric Nephrologist, Manchester, UK; 21 Transplant Nurse, Evelina, London, UK; 22 Consultant Transplant Surgeon, Nottingham, UK; 23 Consultant Paediatric Nephrologist, Leeds, UK

## Introduction

This guideline makes recommendations for immunosuppressive and anti-infective prescribing and monitoring in children and young people (CYP) receiving routine, kidney-only transplants.

**Table 1:** PICO characteristics

Population	Intervention	Comparison	Outcome	Study design
Children (< 18 years) undergoing renal transplantation	Basiliximab, Tacrolimus, Prednisolone, Mycophenolate Mofetil, Azathioprine Valganciclovir Valaciclovir Co-trimoxazole	Any intervention compared with any other or no intervention	Mortality, Hospitalisations, Graft failure, Acute rejection, Infections, Growth.	Randomised controlled trials (RCT), non-randomised studies if adjusted for key confounders (age, health at baseline, co-morbidities).

Current variation in practice of immunosuppressive and anti-infective prescribing in CYP undergoing kidney transplantation impairs assessment of outcomes post transplant and may disadvantage some CYP. Different regimens employed in the UK currently include ‘steroid maintenance’ therapy comprising prednisolone, azathioprine and tacrolimus (PAT); steroid maintenance (PAT) with IL-2 receptor antagonist induction with basiliximab (PAT-B); early steroid withdrawal regimens comprising IL-2 receptor antagonist induction, tacrolimus, mycophenolate mofetil (MMF), and short course of prednisolone (the ‘TWIST’ regimen) and variations of these according to levels of immunological risk, primary disease or co-morbidities (obesity / diabetes / bone disease).

Early steroid withdrawal has been reported to significantly improve growth at 6 months post transplant, with associated improvements in lipid and glucose metabolism [1, 2]. NICE Technology appraisal (TA482, October 2017) recommends that CYP undergoing a first kidney transplant receive IL-2 receptor antagonist, tacrolimus (Adoport®/Modigraf®) and MMF (mycophenolate mofetil) as routine therapy [3]. No recommendations were made for prednisolone or azathioprine, however, some CYP have more adverse events with MMF compared with azathioprine (particularly gastro-intestinal symptoms and bone marrow suppression). Furthermore, there are reports of increased rates of acute rejection in early steroid withdrawal regimens [4]. For these reasons, ISD regimens containing prednisolone or azathioprine continue to be widely used in the UK. In order to improve quality of care and reduce variation in practice, the British Association for Paediatric Nephrology (BAPN), in collaboration with key partners, proposes to undertake a project to develop best practice guidance in the area.

## Summary of recommendations for immunosuppressive (ISD) and anti-infective drug prescribing and monitoring in children and young people receiving routine, initial therapy for kidney-only transplantation

1. We recommend that parents, carers and, where appropriate, the young person should be offered information on steroid maintenance and early steroid withdrawal ISD regimens by health professionals with specialist knowledge in this area. (1D)
2. We recommend that a choice of early steroid withdrawal (short course prednisolone-MMF-tacrolimus-basiliximab, ‘TWIST’) or steroid maintenance (prednisolone-azathioprine-tacrolimus-basiliximab, ‘PAT-B’) ISD regimen should be made jointly by health professionals and parents or carers and, wherever possible, the young person. (1D)
3. We recommend that, for the early steroid withdrawal regimen, the TWIST (short course prednisolone-MMF-tacrolimus-basiliximab) published schedule should be followed per below: (1D)

Basiliximab should be administered 2 h before transplant surgery (d0) and after 4 days (d4) at the following weight-banded doses:

- ≥ 35 kg: 20 mg.
- < 35 kg: 10 mg.

Prednisolone should be prescribed per below (mg/m2 once daily):

**Table:** 

Day of transplant (d0):	*(methylprednisolone - see Q5)*
Day 1 post transplant (d1):	60 (maximum dose 60 mg)
Day 2 post transplant (d2):	40 (maximum dose 40 mg)
Day 3 post transplant (d3):	30 (maximum dose 30 mg)
Day 4 post transplant (d4):	20 (maximum dose 20 mg)
Day 5 onwards: (d5):	0

Tacrolimus should be prescribed (initial dosing) at 0.15 mg/kg twice daily with a maximum initial dose of 5 mg twice daily.

Mycophenolate mofetil (MMF) should be prescribed as 600 mg / m^2^ (maximum 1 g) twice daily on days 0–14, then 300 mg / m^2^ twice daily from day 15 onwards.

4. We recommend that, for the PAT-B (prednisolone-azathioprine-tacrolimus-basiliximab) regimen:

Basiliximab should be administered 2 h before transplant surgery (d0) and after 4 days (d4) at the following weight-banded doses:

- ≥ 35 kg: 20 mg.
- < 35 kg: 10 mg.

Prednisolone should be prescribed per below (mg/m^2^ once daily):

**Table:** 

Day of transplant (d0):	*methylprednisolone (see Recommendation 5)*
Day 1–2 post transplant:	60 (maximum dose 60 mg)
Day 3–7 post transplant:	40 (maximum dose 40 mg)
Day 8–14 post transplant:	30 (maximum dose 30 mg)
Day 15–21 post transplant:	20 (maximum dose 20 mg)
Day 22–28 post transplant:	10 (maximum dose 10 mg)
Day 29–90 post transplant:	10 (maximum dose 10 mg) on alternate days
Day 91 post transplant->	5 (maximum dose 5 mg) on alternate days

Azathioprine should be prescribed at 2 mg/kg daily from day 0 (day of transplant) onwards.

Tacrolimus should be prescribed (initial dosing) at 0.15 mg/kg twice daily with a maximum initial dose of 5 mg twice daily.

5. We recommend that, for children and young people receiving either PAT-B or TWIST regimens, Methylprednisolone 600 mg/m^2^ (maximum dose 500 mg) should be given at induction or reperfusion on the day of transplant (day 0) (1D).
6. We recommend that, for children and young people receiving either PAT-B or TWIST regimens, tacrolimus should be commenced on the day of transplant (day 0) for living and deceased donor transplants (1D).
7. We recommend that, for children and young people receiving either PAT-B or TWIST regimens, Target ranges for tacrolimus 12 h trough levels should be: (1D)
  8–12 ng/ml for months 1 and 2 post transplant.
  5–8 ng/ml for months 3 to 12 post transplant.
  Beyond the first year, tacrolimus levels may be individualized.
8. We recommend that children and young people should receive pneumocystis prophylaxis with co-trimoxazole for 6 months post-transplant.
9. We recommend that children and young people should receive prophylaxis with valganciclovir for at least 3 months post-transplant if the donor is CMV positive and recipient CMV negative (D + R-). (1D)
10. We recommend that children and young people should be monitored for CMV viral load at least monthly for 12 months post-transplant if either donor or recipient are CMV positive (CMV D + R- / CMV D-R+ / CMV D + R+). (1D)

## Summary of audit measures for immunosuppressive and anti-infective drug prescribing and monitoring in children and young people receiving routine, initial therapy for kidney-only transplantation

1. Proportion of parents or carers of CYP undergoing renal transplantation offered information on steroid maintenance and early steroid withdrawal ISD regimens by health professionals with specialist knowledge in this area.
2. Proportion of CYP prescribed an early steroid withdrawal regimen post renal transplant, receiving medications per the TWIST (short course prednisolone-MMF-tacrolimus-basiliximab) published schedule as detailed in recommendation 3.
3. Proportion of CYP prescribed a steroid maintenance regimen post renal transplant, receiving medications per the PAT-B (prednisolone-azathioprine-tacrolimus-basiliximab) regimen published schedule as detailed in recommendation 4.
4. Proportion of CYP receiving either PAT-B or TWIST regimens receiving Methylprednisolone 600 mg/m^2^ (maximum dose 500 mg) at induction or reperfusion on the day of transplant.
5. Proportion of CYP receiving either PAT-B or TWIST regimens, commencing tacrolimus on the day of transplant (day 0; living and deceased donor transplants).
6. Proportion of CYP receiving either PAT-B or TWIST regimens maintaining tacrolimus 12 h trough levels as detailed in recommendation 7.
7. Proportion of CYP offered pneumocystis prophylaxis with co-trimoxazole for 6 months post-transplant.
8. Proportion of CYP offered prophylaxis with valganciclovir for at least 3 months post-transplant where the donor is CMV positive and recipient CMV negative (D + R-).
9. Proportion of CYP undergoing monitoring for CMV viral load for 12 months post-transplant if either donor or recipient are CMV positive (CMV D + R- / CMV D-R+ / CMV D + R+).

## Summary of research recommendations for immunosuppressive and anti-infective drug prescribing and monitoring in children and young people receiving routine, initial therapy for kidney-only transplantation

1. In children and young people receiving initial therapy for routine, kidney only transplantation, is early steroid withdrawal associated with improved outcomes compared with steroid maintenance therapy?

## Rationale for clinical practice recommendations for immunosuppressive and anti-infective drug prescribing and monitoring in children and young people receiving routine, initial therapy for kidney-only transplantation

1. **We recommend that parents, carers and, where appropriate, the young person should be offered information on steroid maintenance and early steroid withdrawal ISD regimens by health professionals with specialist knowledge in this area. (1D)**

### Audit measure

Proportion of parents or carers of CYP undergoing renal transplantation offered information on steroid maintenance and early steroid withdrawal ISD regimens by health professionals with specialist knowledge in this area.

### Rationale

No relevant studies were identified for this review question, however, NICE guidance on patient experience in adult NHS services recommends that patients should be provided with information, and the support they need to promote their active participation in care and self-management. This should include information about relevant treatment options and services that they are entitled to, even if these are not provided locally [5]. There was 91% agreement with this recommendation in the Delphi consensus process (consensus reached).

2. **We recommend that a choice of early steroid withdrawal (short course prednisolone-MMF-tacrolimus-basiliximab, ‘TWIST’) or steroid maintenance (prednisolone-azathioprine-tacrolimus-basiliximab, ‘PAT-B’)) ISD regimen should be made jointly by health professionals and parents or carers and, wherever possible, the young person. (1D)**

### Rationale

No relevant studies were identified for this review question, however, as discussed above, NICE guidance on patient experience in adult NHS services recommends that patients should be provided with information, and the support they need to promote their active participation in care and self-management. This should include information about relevant treatment options and services that they are entitled to, even if these are not provided locally [5]. There was 81% agreement with this recommendation in the Delphi consensus process (consensus reached), however, both Health professionals and lay representatives expressed concern that the issue of ISD regimens is so complex that it is the responsibility of health care professionals to a make clear recommendation with regard to the preferred regimen in each particular case, as the need for one or other regimens will vary in different clinical situations.

3. **We recommend that, prescribing for the early steroid withdrawal regimen, should be based on the TWIST (short course prednisolone-MMF-tacrolimus-basiliximab) published schedule per below: (1D)**

Basiliximab should be administered 2 h before transplant surgery (d0) and after 4 days (d4) at the following weight-banded doses:

- ≥ 35 kg: 20 mg.
- < 35 kg: 10 mg.

Prednisolone should be prescribed per below (mg/m2 once daily):

**Table:** 

Day of transplant (d0):	*(methylprednisolone - see Q5)*
Day 1 post transplant (d1):	60 (maximum dose 60 mg)
Day 2 post transplant (d2):	40 (maximum dose 40 mg)
Day 3 post transplant (d3):	30 (maximum dose 30 mg)
Day 4 post transplant (d4):	20 (maximum dose 20 mg)
Day 5 onwards: (d5):	0

Tacrolimus should be prescribed (initial dosing) at 0.15 mg/kg twice daily with a maximum initial dose of 5 mg twice daily.

Mycophenolate mofetil (MMF) should be prescribed as 600 mg / m2 (maximum 1 g) twice daily on days 0–14, then 300 mg / m2 twice daily from day 15 onwards.

### Audit measure

Proportion of CYP prescribed an early steroid withdrawal regimen post renal transplant, receiving medications per the TWIST (short course prednisolone-MMF-tacrolimus-basiliximab) published schedule as detailed above.

### Rationale

There was 83% agreement with this recommendation in the Delphi consensus process (consensus reached).

Early steroid withdrawal is reported to be associated with better growth and a reduced incidence of new onset diabetes post transplant in CYP undergoing renal transplantation [1, 2]. NICE Technology appraisal (TA482, October 2017) recommends that CYP undergoing a first renal transplant receive IL-2 receptor antagonist, tacrolimus (Adoport®/Modigraf®) and MMF (mycophenolate mofetil) as routine therapy [2]. The NICE guidance does not, however, specify dosing of medication and members of the guideline committee were in agreement that prednisolone prescribing should be standardised per the published protocol in the TWIST study [1], whilst tacrolimus prescribing should be in line with recommendations in the British National Formulary for Children (BNFC) [6]. Due to concerns about higher concentrations of tacrolimus in adolescents, the committee agreed to propose an upper dose limit of 5 mg at initiation, as is undertaken in some adult units, with subsequent dosing being directed by tacrolimus monitoring.

4. **We recommend that, for the PAT-B (prednisolone-azathioprine-tacrolimus-basiliximab) regimen: (1D)**

Basiliximab should be administered 2 h before transplant surgery (d0) and after 4 days (d4) at the following weight-banded doses:

- ≥ 35 kg: 20 mg.
- < 35 kg: 10 mg.

Prednisolone should be prescribed per below (mg/m2 once daily):

**Table:** 

Day of transplant (d0):	*methylprednisolone (see Recommendation 5)*
Day 1–2 post transplant:	60 (maximum dose 60 mg)
Day 3–7 post transplant:	40 (maximum dose 40 mg)
Day 8–14 post transplant:	30 (maximum dose 30 mg)
Day 15–21 post transplant:	20 (maximum dose 20 mg)
Day 22–28 post transplant:	10 (maximum dose 10 mg)
Day 29–90 post transplant:	10 (maximum dose 10 mg) on alternate days
Day 91 post transplant->	5 (maximum dose 5 mg) on alternate days

Azathioprine should be prescribed at 2 mg/kg daily from day 0 (day of transplant) onwards. Tacrolimus should be prescribed (initial dosing) at 0.15 mg/kg twice daily with a maximum initial dose of 5 mg twice daily.

### Audit measure

Proportion of CYP prescribed a steroid maintenance regimen post renal transplant, receiving medications per the PAT-B (prednisolone-azathioprine-tacrolimus-basiliximab) regimen published schedule as detailed above.

### Rationale

There was 77% agreement with this recommendation in the Delphi consensus process (consensus reached).

NICE Technology appraisal (TA482, October 2017) recommends that CYP undergoing a first renal transplant receive IL-2 receptor antagonist, tacrolimus (Adoport®/Modigraf®) and MMF (mycophenolate mofetil) as routine therapy [3]. No recommendations were made for prednisolone or azathioprine, however, some CYP have more adverse events with MMF compared with azathioprine, particularly gastro-intestinal symptoms and bone marrow suppression. Furthermore, recent studies have reported an increased incidence of acute rejection in adults and children receiving steroid avoidance and withdrawal drug regimens after kidney transplantation. Authors of a systematic review in 2016 concluded that long-term consequences of steroid avoidance and withdrawal remain unclear because prospective long-term studies have not been conducted [4]. For these reasons, committee members agreed that a steroid maintenance regimen should continue to be offered as one of 2 ISD regimens as initial therapy to CYP undergoing routine, kidney-only transplants.

The committee identified marked variability in prednisolone prescribing in CYP receiving ‘steroid maintenance’ therapy in the UK and some variability in prescribing IL-2 receptor antagonist induction between UK centres when reviewing centre protocols. In order to reduce variations in practice, the committee agreed to propose standardisation of prednisolone dosing and the use of IL-2 receptor antagonist induction for all CYP undergoing routine, kidney-only transplants. The committee agreed that the addition of IL-2 receptor antagonist induction may allow a reduction in initial steroid dosing and proposed the steroid reduction schedule described above. The committee also agreed that tacrolimus prescribing should be in line with recommendations in the British National Formulary for Children (BNFC) [6]. Due to concerns about higher concentrations of tacrolimus in adolescents, the committee agreed to propose an upper dose limit of 5 mg at initiation, as is undertaken in some adult units, with subsequent dosing being directed by tacrolimus monitoring.

5. **We recommend that, for children and young people receiving either PAT-B or TWIST regimens, Methylprednisolone 600 mg/m**^**2**^**(maximum dose 500 mg) should be given at induction or reperfusion on the day of transplant (day 0) (1D)**

### Audit measure

Proportion of CYP receiving either PAT-B or TWIST regimens receiving Methylprednisolone 600 mg/m^2^ (maximum dose 500 mg) at induction or reperfusion on the day of transplant.

### Rationale

No relevant studies assessing precise dosing for Methylprednisolone in CYP undergoing routine kidney-only transplantation were identified for this review question. There was 78% agreement with this recommendation (statement) by the Delphi panelists in the 2nd round (consensus reached). Some panelists rejected the statement in the 1st round due to a concern about the timing of administration, with some clinicians preferring to administer the methyl-prednisolone at reperfusion rather than induction. The committee agreed to include ‘at induction or reperfusion’ in the recommendation. It was noted in both rounds of the Delphi consensus process that a number of panelists had raised concerns that the recommended upper dose of 500 mg may be too low, whilst one panelist expressed concerns about Methylprednisolone being used at all in the absence of evidence, and in the face of improvements of techniques to assess histocompatibility and improvements in immunosuppression. The committee agreed that it was reasonable to make this recommendation, retaining the recommended upper dose on the basis of the overall support received.

6. **We recommend that, for children and young people receiving either PAT-B or TWIST regimens, tacrolimus should be commenced on the day of transplant (day 0) for living and deceased donor (DD) transplants (1D)**

### Audit measure

Proportion of CYP receiving either PAT-B or TWIST regimens, commencing tacrolimus on the day of transplant (day 0; living and deceased donor transplants).

### Rationale

No relevant studies were identified for this review question. There was 85% agreement with this recommendation in the Delphi consensus process (consensus reached). The committee noted the existing variation in practice across the UK, with some centres commencing tacrolimus up to 48 h prior to living donor transplant. In the absence of evidence of improved outcomes in CYP receiving tacrolimus prior to the day of transplant, the committee agreed to recommend that time of commencement of tacrolimus for CYP receiving living donor transplants should be the same as that for CYP receiving deceased donor (DD) transplants.

7. **We recommend that, for children and young people receiving either PAT-B or TWIST regimens, Target ranges for tacrolimus 12 h trough levels should be: (1D)**

8–12 ng/ml for months 1 and 2 post transplant.

5–8 ng/ml for months 3 to 12 post transplant.

Beyond the first year, tacrolimus levels may be individualized.

### Audit measure

Proportion of CYP receiving either PAT-B or TWIST regimens maintaining tacrolimus 12 h trough levels as detailed above.

### Rationale

No relevant studies were identified for this review question. There was 83% agreement with this recommendation in the Delphi consensus process.

The committee identified variability in tacrolimus target levels in CYP undergoing renal transplantation in the UK. In order to reduce variation in practice, the committee agreed to propose standardisation of tacrolimus target levels for CYP undergoing routine, kidney-only transplants. The committee agreed that the addition of IL-2 receptor antagonist induction may allow a reduction in tacrolimus target levels, noting that tacrolimus toxicity may be more problematic than acute rejection. The committee also agreed that there will need to be flexibility to alter thresholds before 12 months in those patients showing evidence of tacrolimus toxicity.

8. **We recommend that children and young people should receive pneumocystis prophylaxis with co-trimoxazole for 6 months post-transplant. (1D)**

### Audit measures

1. Proportion of CYP offered pneumocystis prophylaxis with co-trimoxazole for 6 months post transplant where the donor is CMV positive and recipient CMV negative (D + R-)
2. Proportion of CYP receiving co-trimoxazole developing neutropaenia.

### Rationale

No relevant studies were identified for this review question. There was 86% agreement with this recommendation in the Delphi consensus process (consensus reached), however, some panelists expressed concern about the proposed duration of 6 months, due to the potential for side effects including bone marrow suppression and nephrotoxicity [6]. The committee agreed that dose adjustment or cessation due to tolerability would be at clinicians’ discretion.

9. **We recommend that children and young people should receive prophylaxis with valganciclovir for at least 3 months post transplant if the donor is CMV positive and recipient CMV negative (D + R-). (1D)**

### Audit measures

1. Proportion of CYP offered valganciclovir prophylaxis where the donor is CMV positive and recipient CMV negative (D + R-)
2. Proportion of CYP receiving valganciclovir becoming neutropaenic

### Rationale

Valganciclovir has been widely used for cytomegalovirus (CMV) prophylaxis in solid-organ transplant recipients. Limited evidence suggests that 6 months of prophylaxis may be associated with a lower rate of late-onset disease than 3 month regimens in CYP receiving valganciclovir [7], however, the committee agreed to propose a recommendation for ‘at least 3 months’ of valganciclovir due to concerns about its significant side effect profile (infection, diarrhoea, leukopenia, neutropenia) [8].

There was 93% agreement with this recommendation in the Delphi consensus process (consensus reached).

10. **We recommend that children and young people should be monitored for CMV viral load at least monthly for 12 months post transplant if either donor or recipient are CMV positive (CMV D + R- / CMV D-R+ / CMV D + R+). (1D)**

### Audit measures

1. Proportion of CYP undergoing monitoring for CMV viral load for 12 months post transplant if either donor or recipient are CMV positive (CMV D + R- / CMV D-R+ / CMV D + R+).
2. Proportion of CYP receiving valganciclovir becoming neutropaenic.

### Rationale

No relevant studies were identified for this review question, however, the incidence of CMV disease and / or viraemia in has been reported in 41% of CMV positive donor and CMV negative recipient (D+/R-) and 24% of CMV positive recipients (D+/R+ and D−/R+) [9]. The committee noted the existing variation in practice across the UK and agreed to recommend CMV viral load monitoring on a monthly basis in all CYP post-transplant. Members of the committee agreed that management of CMV viraemia would be dictated by local practice.

There was 79% agreement with this recommendation in the Delphi consensus process (consensus reached).

## Rationale for research recommendations for immunosuppressive and anti-infective drug prescribing and monitoring in children and young people receiving routine, initial therapy for kidney-only transplantation

1. **In children and young people receiving initial therapy for routine, kidney only transplantation, is early steroid withdrawal associated with improved outcomes compared with steroid maintenance therapy?**

### Rationale

Early steroid withdrawal has been reported to significantly improve growth at 6 months post-transplant, with associated improvements in lipid and glucose metabolism [1, 2], however, some CYP have more adverse events with MMF compared with azathioprine (particularly gastro-intestinal symptoms and bone marrow suppression). There are reports of increased rates of acute rejection in early steroid withdrawal regimens [4] and long term outcome data are lacking. Furthermore, it is conceivable that the overall reduction in steroid dosing achieved through standardisation of prednisolone prescribing and use of IL-2 receptor antagonist in all UK centres may be associated with improved growth in CYP receiving steroid maintenance treatment.

## Information for parents and carers of children and young people receiving routine, kidney-only transplantation

Around 150 children and young people (CYP) receive a kidney transplant every year in the UK. These CYP require medications to prevent the body from ‘rejecting’ the kidney transplant, by suppressing (‘turning down’) the immune system. These treatments have important side effects, including infections, diabetes, diarrhoea and nausea and poor growth. Different treatment regimens have different side effects. There is some variation across the UK in how health professionals prescribe these treatments.

This guideline is intended to help CYP undergoing kidney transplantation and their families by making sure that:

- Health professionals with specialist knowledge in kidney transplantation offer you information on the available treatments and their side effects.
- Prescribing of immunosuppression or ‘anti-rejection’ treatment is standardised across the UK in 2 agreed ‘regimens’ to reduce variation in practice.

## Data Availability

Not applicable.

## Abbreviations

BAPN: British Association for Paediatric Nephrology
BNFC: British National Formulary for Children
CMV: Cytomegalo virus
CMV D+R-: CMV donor positive, recipient negative
CMV D-R+: CMV negative, recipient positive
CMV D+R+: CMV donor positive, recipient positive
CYP: Children and young people
DD: Deceased donor
IL-2: Interleukin-2
ISD: Immunosuppressive drug
MMF: Mycophenolate mofetil
NICE: National Institute for Health and Care Excellence
PAT: Prednisolone, azathioprine and tacrolimus
PAT-B: Prednisolone, azathioprine, tacrolimus and induction with basiliximab
PICO: Population, Intervention, Comparator, Outcome
TWIST: Short course prednisolone, mycophenolate mofetil, tacrolimus, basiliximab
